# Using psychological theory to inform methods to optimize the implementation of a hand hygiene intervention

**DOI:** 10.1186/1748-5908-7-77

**Published:** 2012-08-28

**Authors:** Veronique M Boscart, Geoff R Fernie, Jae H Lee, Susan B Jaglal

**Affiliations:** 1 Schlegel-University of Waterloo Research Institute for Aging (RIA) & School of Health & Life Sciences and Community Services, Conestoga College Institute of Technology and Advanced Learning, Kitchener, ON, Canada; 2 Toronto Rehabilitation Institute, Toronto, ON, Canada; 3 Department of Surgery, University of Toronto, Toronto, ON, Canada; 4 Department of Physical Therapy, University of Toronto, Toronto, ON, Canada

**Keywords:** Hand hygiene, Knowledge translation, Compliance, Behaviour change, Electronic monitoring

## Abstract

**Background:**

Careful hand hygiene (HH) is the single most important factor in preventing the transmission of infections to patients, but compliance is difficult to achieve and maintain. A lack of understanding of the processes involved in changing staff behaviour may contribute to the failure to achieve success. The purpose of this study was to identify nurses’ and administrators’ perceived barriers and facilitators to current HH practices and the implementation of a new electronic monitoring technology for HH.

**Methods:**

Ten key informant interviews (three administrators and seven nurses) were conducted to explore barriers and facilitators related to HH and the impact of the new technology on outcomes. The semi structured interviews were based on the Theoretical Domains Framework by Michie *et al.* and conducted prior to intervention implementation. Data were explored using an inductive qualitative analysis approach. Data between administrators and nurses were compared.

**Results:**

In 9 of the 12 domains, nurses and administrators differed in their responses. Administrators believed that nurses have insufficient knowledge and skills to perform HH, whereas the nurses were confident they had the required knowledge and skills. Nurses focused on immediate consequences, whereas administrators highlighted long-term outcomes of the system. Nurses concentrated foremost on their personal safety and their families’ safety as a source of motivation to perform HH, whereas administrators identified professional commitment, incentives, and goal setting. Administrators stated that the staff do not have the decision processes in place to judge whether HH is necessary or not. They also highlighted the positive aspects of teams as a social influence, whereas nurses were not interested in group conformity or being compared to others. Nurses described the importance of individual feedback and self-monitoring in order to increase their performance, whereas administrators reported different views.

**Conclusions:**

This study highlights the benefits of using a structured approach based on psychological theory to inform an implementation plan for a behavior change intervention. This work is an essential step towards systematically identifying factors affecting nurses’ behaviour associated with HH.

## Background

Transmission of infections within healthcare institutions presents a significant threat to the health of patients and staff [1]. It has been estimated that there are 2 million hospital-acquired infections (HAIs) per year globally, affecting 10% of hospitalized patients [2,3]. In the United States, HAIs are estimated to cause 90,000 deaths annually and result in $5.7 billion in excess healthcare costs [4,5]. In Canada, HAIs affect 220,000 patients, resulting in 8,000 deaths per year [6]. Most HAIs must be treated with antibiotics, resulting in increases in antibiotic-resistant organisms, lengthened hospital stays, additional surgical procedures, inefficiency in hospital systems, disability, and sometimes death.

Careful hand hygiene (HH) performed by healthcare staff is the single most important factor in preventing the transmission of pathogens [7-9]. Research has indicated that up to 50% of HAIs could be avoided with improvements in HH compliance [3,8,9]. Therefore, HH is recommended as a routine best practice for all healthcare staff-patient interactions [3,8]. Several evidence-based guidelines and interventions have been developed to enhance HH compliance,[10,11] and most healthcare organizations have invested significant resources in the training and education of their staff. In spite of this, improved compliance with HH guidelines is difficult to achieve and maintain, partially because education alone does not translate into practice change in the demanding clinical environments in which staff practice [9]. Multiple studies have documented HH compliance rates to be suboptimal, with a mean observed rate of 40% [2,12].

To address this concerning lack of professional practice, a variety of promotional strategies and interventions have been studied, yet none have demonstrated significant sustained improvements in HH compliance [3,12-14]. Multimodal interventions, such as those that include both problem-based and task-oriented HH education, ongoing HH audits, and HAI surveillance programs, do improve HH compliance rates but have failed to be sustainable because they require substantial ongoing costs for educators, auditors, and administrators [13]. Rosenthal *et al.*[15] reported an atypical improvement in HH compliance as a result of an education, training, and performance feedback program, with an increase in compliance from 23% to 65%. The authors claimed a sustained improvement, yet significant ongoing effort in the form of observation twice a week over an 18-month period was used to maintain this change.

Given the current challenges to promote HH, an electronic monitoring system, reminding staff to wash their hands when necessary, may facilitate compliance [16]. Swoboda *et al.*[17] evaluated an electronic monitoring system using light beams and motion detectors at the threshold of each room, with additional sensors detecting the use of sinks, soap, and alcohol gel dispensers. A voice-prompting system instructed the staff to wash their hands if they had not done so before exiting the room. HH compliance improved by 41%, and infection rates decreased by 48%; however, the authors concluded that compliance was “at best, fair, even in a population of professionals who understand the importance (p. 363). No data were collected on staff’s responses to being monitored, nor on the acceptability of the system [17].

The limited impact of these described strategies and interventions may be due in part to a failure to identify those factors influencing staff’s HH behavior and subsequent lack of incorporation of these findings into intervention designs and the delivery of the intervention. There is some research available indicating that several barriers and facilitators do influence HH compliance, [3,12-14] yet this information has not been translated in intervention design and implementation.

### A new technology to enhance hand hygiene

A team of researchers has developed a novel technology that allows electronic HH monitoring and prompting of staff [18,19]. The system consists of individual worn badges that communicate with sensors installed at room entrances and hand wash stations throughout the unit. Staff entering or leaving a room are prompted to wash their hands if they have not done so previously. To the best of the authors’ knowledge, no other systems with similar capabilities currently exist. A pilot study exploring the acceptability of this electronic monitoring system (EMS) indicated a favourable response by staff, [20] and a larger study evaluating the efficacy of the system and the sustainability of resulting improvements in HH compliance has been funded. Nurses on a 50-bed unit will be using the EMS for 12 months, and qualitative and quantitative data will be collected. This novel technology has the potential to improve and sustain compliance beyond what has been accomplished previously [21]. However, as with any intervention aimed at changing professionals’ behaviour, its effectiveness is sensitive to context [22,23].

A clear understanding of why staff do or do not change their individual behavior is essential in order to guide intervention design [22,24]. More specifically, to justify program implementation of the EMS, it will be of critical importance to understand the perceived barriers and facilitators underlying individual nurses’ compliance. Furthermore, this information is crucial to design further interventions, disseminate results, and help governments and others to integrate and update HH compliance standards and policies [25]. The purpose of this study was therefore to explore barriers and facilitators to current HH practices and the implementation of the EMS intervention to improve HH practice from the perspective of nurses and administrators. Nurses represent the largest proportion of healthcare staff that have direct contact with patients and are, therefore, an important target group of any HH intervention. The findings of this study will be used to inform implementation of the EMS.

## Methods

### Design

A qualitative design using semi structured key informant interviews was used to collect data. Key informant interviews consist of qualitative in-depth interviews from a range of people who have firsthand knowledge about the phenomena in the practice setting. These experts, with their particular knowledge and understanding, can provide insight on the nature of problems and give recommendations for solutions. For this specific study, the authors chose to involve the director of care, the unit manager, an infection control specialist, and nurses as key informants.

In determining the approach to gather the information needed, the authors chose the Theoretical Domains Framework (TDF) of behaviour change developed by Michie *et al.*, [26] as the domains aligned well with our aim to explore barriers and facilitators of a HH behavior change.

### Setting and participants

Semi structured in-depth interviews were undertaken to identify staff’s perceived barriers and facilitators to current HH practices and the implementation of the EMS. All nursing staff employed on the intervention unit were invited to participate in the study. Inclusion criteria included being a part-time or full-time employee on the designated unit and providing direct care. In addition, the infection control specialist and the director of care for the facility and the unit manager responsible for the intervention unit were invited. All potential participants were contacted and received an information letter about the study. If they agreed to receive more information, one of the investigators (VMB) met with them and further explained the study and invited them to participate. Interested employees were then asked to sign a consent form.

Individual interviews were conducted with a convenience sample of three healthcare administrators (one unit manager, one director of care, and one infection control specialist) and eight nurses. There is no firm and fast rule regarding the appropriate sample size in interpretive descriptive research [27]; however, Thorne [28] notes that the vast majority of studies using this approach are likely to be relatively small. In addition, the predetermined designation of the intervention unit limited the sample size; the facility employs only one director of care and three infection control specialists, and the intervention unit employs 24 nurses, further limiting the comparison between nurses’ responses and those of the administrators. To address these issues and to determine an appropriate sample size, the authors applied the principles for deciding saturation in theory-based interview studies, outlined by Francis and colleagues [27]. A minimum sample size for initial analysis was determined to be six nurses and three administrators. Subsequently, two more interviews with nurses were conducted without new ideas emerging. The authors were unable to recruit additional administrators, as there were none available.

### Ethics

All recruitment and data collection procedures were approved by the facility’s Research Ethics Board prior to the start of the study. Given the potential to identify the individual administrators as the director of care, infection control specialist, or the unit manager, the authors decided to report their data as grouped responses, rather than individual data. No personal information was recorded. Only the researchers had access to the data.

### Interview guide

The TDF of behavior change describes a theoretical domain interview (TDI) [26] with 12 possible domains that can facilitate or hinder successful intervention implementation: knowledge, skills, social/professional role and identity, beliefs about capabilities, beliefs about consequences, motivation and goals, memory and attention and decision processes, environmental context and resources, social influences, emotion, behavioral regulation, and nature of the behaviours (Table 1). All 12 of these domains were of interest, so the TDI guide was adapted to our study. For each of the 12 possible domains that could act as facilitators or barriers to current HH practices and a successful HH intervention implementation, the authors developed several interview questions. These questions explored factors that might influence nurses’ behavior change related to HH in general and the specific EMS (Table 1).

**Table 1:** Behavioral-change domains and some interview questions to explore behaviour change

**Behavioral-change domains**	**Some interview questions to explore behaviour change**
Knowledge	Can you describe the guidelines to perform proper HH?
	Can you discuss when to perform HH?
	Can you describe why you should be performing HH?
	Can you describe how the EMS works?
	Do you know what information the EMS can collect?
Skills	Can you explain the proper procedure of performing HH?
	How easy or difficult is it to perform HH on your unit?
	Can you describe how to use the EMS?
	Do you know how to respond when the EMS reminds you?
Social/professional role and identity (self-standards)	What role will the EMS play in enhancing HH?
	Do you think the EMS should determine how you perform HH?
	Do you feel that the guidelines for performing HH with the EMS are congruent with your professional standards of practice?
	Should proper HH be practiced at all levels of staff?
Beliefs about capabilities (self-efficacy)	Difficult or easy is it for you to maintain proper HH?
	What problems have you encountered when trying to practice proper HH?
	What would help you to increase HH compliance?
	How confident are you that you can increase compliance with the barriers and difficulties you face?
	How well equipped and comfortable do you feel in increasing your level of HH compliance? When using the EMS?
	How capable do you feel in maintaining increased compliance with HH? When using the EMS?
	How well will this EMS record your HH?
Beliefs about consequences (anticipated outcomes/ attitudes)	Does HH play an important role in your current practice? For yourself? For your patients? Can you explain why?
	Do you believe that this EMS will play an important role in your practice?
	Do you foresee any positive or negative outcomes of increased HH compliance on patient outcomes? Staff outcomes? Do you foresee these outcomes/consequences as long term or short term?
	Do you foresee a negative consequence of using the EMS? For patient outcomes? Staff outcomes?
	What do you think will happen if HH compliance is not increased in terms of patient outcomes? Staff outcomes? Do you think these are short- or long-term consequences?
	How will you feel if you are able to increase HH compliance? How will you feel if you do not?
Motivation and goals (intention)	Would you like to increase your HH compliance?
	Do you feel a need to increase your HH compliance?
	What are your reasons for increasing your HH compliance?
	Is there any aspect of your HH performance that you could improve on? Frequency, activity related?
	Are there other things that you would like to achieve that might interfere with increasing your HH compliance?
	Are there incentives to increasing HH compliance? If so, what are they?
	Are there incentives to use the EMS? If so, what are they?
Memory, attention, and decision processes	Do you usually perform HH? How often on a regular shift?
	Do you consciously think and make the decision to wash your hands?
	What factors influence that decision? Type of care activity? Type of patient? Time?
	How much attention do you have to pay to perform HH?
	Do you remember to perform HH? How?
	Do you think the reminder system in the EMS will enhance your HH?
	Can you think of times where you might not perform HH, such as competing tasks or time constraints?
Environmental context and resources (environmental constraints)	Where do you currently disinfect your hands?
	Have you used a wearable alcohol dispenser device? How does this impact your HH performance?
	To what extent do physical or resource factors, such as the availability and functioning of wall units and technology, facilitate or hinder performing HH?
	Do you think necessary resources are available for staff to increase HH compliance?
	Do you believe that the EMS will enhance your HH performance?
Social influences (norms)	Does HH play an important role on your unit? Can you explain why?
	Do you believe that nursing staff on this unit are washing their hands when necessary?
	To what extent do social influences facilitate or hinder performing HH? Social influence from your peers? Managers? Other professional groups? Patients? Relatives?
	Do you believe that there will be social influences from your peers to use the EMS? Managers? Patients? Other groups?
	Will you or have you ever observed others performing HH?
	Do you have role models in performing HH?
Emotion	Does performing HH elicit an emotional response? If so, what?
	To what extent will emotional factors facilitate or hinder your HH?
	Do you believe that emotional factors will influence the use of the EMS?
Behavioral regulation	What initial steps need to be taken to improve HH compliance/use the EMS on an individual level?
	How about on an organizational level?
	Can you think of any procedures that would encourage increased HH compliance/use of EMS?
Nature of the behaviours	How will the EMS increase HH compliance?
	Who needs to work differently for this to occur? When? Where?
	How do you know whether increased HH compliance has occurred?
	What do you currently do in terms of performing HH?
	Is this a new or existing behavior that needs to become a habit?
	Can the context be used to prompt you to perform HH? (prompts: layout, reminders, equipment)
	How long do you think the changes are going to take?

*HH* = hand hygiene; *EMS* = electronic monitoring system.

### Coding reliability

Each interview followed the same protocol to ensure quality control and started with broad, open-ended questions followed by an increasing focus on specific issues. The interviews were conducted in a private setting using a semistructured guide, audiotaped, and transcribed verbatim. Subsequently, each transcript was reviewed by the interviewer to ensure accuracy and inclusion of expressive detail [29].

### Transcript analysis

Data were extracted by employing an inductive, interactive, comparative process that allows ideas or categories to arise from the data [30]. First, all of the interview data were reviewed and broken down into the TDI theoretical domains. Next, within each theoretical domain, discrete concepts were identified to allow comparisons for similarities and differences (Table 2). An initial coding scheme was developed to permit connections between concepts. The final step consisted of systematically relating core categories to other categories to extract barriers and facilitators that could influence HH behaviour and the intervention implementation.

**Table 2:** Behavioral-change domains and descriptive codes

**Behavioral-change domains**	**Descriptive codes**
Knowledge	Description of information and knowledge as facilitators to performing HH and using the EMS.
Skills	Description of skills and competencies as facilitators to performing HH and using the EMS.
Social/professional role and identity (self-standards)	Description of the staff’s role and identity on the unit as facilitators to performing HH and using the EMS.
Beliefs about capabilities (self-efficacy)	Description of perceived self-efficacy and control of behaviors as facilitators to performing HH and using the EMS.
Beliefs about consequences (anticipated outcomes/ attitudes)	Description of beliefs about the consequences as facilitators to performing HH and using the EMS.
Motivation and goals (intention)	Description of motivation and goals as facilitators to performing HH and using the EMS.
Memory, attention, and decision processes	Description of memory, attention, and decision processes as facilitators to performing HH and using the EMS.
Environmental context and resources (environmental constraints)	Description of availability and accessibility of resources as facilitators to performing HH and using the EMS.
Social influences (norms)	Description of social influence and the role of the healthcare team as facilitators to performing HH and using the EMS.
Emotion	Description of emotion (stress, fear, burnout, or positive or negative emotional responses) as a facilitator to performing HH and using the EMS.
Behavioral regulation	Description of behavioral regulation as a facilitator to performing HH and using the EMS.
Nature of the behaviours	Description of the nature of the behavior as a facilitator to performing HH and using the EMS.

*HH* = hand hygiene; *EMS* = electronic monitoring system.

Two evaluators independently reviewed interview transcripts to identify key words, phrases, and concepts used by the participants to enhance accuracy of the analysis. Subsequently, they compared and contrasted codes emerging from the data to ensure consistency in the definitions and interpretations of the codes. All data were coded electronically according to standard qualitative coding techniques [28]. NVivo 9 (QSR International, Cambridge, MA, USA), an advanced storage-code-and-retrieval software program, facilitated the organization and analysis of the data. To ensure the reliability of the analyses, an audit trail was used to maintain records and encourage reflexivity of data collection and analysis procedures [31].

## Results

Participants had no difficulty commenting on each of the different theoretical domains. The data provide valuable insights into our understanding of potential barriers and facilitators within each domain to current HH practices and to implementing the EMS intervention in clinical practice. A summary of the key findings is presented below.

### Knowledge

The knowledge domain contains the information the nurses have in regards to HH, the rationale about HH compliance, the scientific evidence supporting HH, and the procedural knowledge to perform HH and explores if these act as facilitators with regards to HH. This domain also includes the participants’ understanding and perceived rationale behind the EMS system, which could potentially facilitate the intervention. Our main finding for this domain was that the administrators (ADM), consisting of the director of care, the unit manager, and the infection control specialist, all believed that the staff have some understanding and knowledge relevant to the implementation of the EMS, yet this knowledge is not sufficient. Nurses, on the other hand, were confident they have the required knowledge to perform HH and use the EMS. Examples of this included the following:

"I would assume that people that work within our program should be well advised. (ADM 1)"

"We have enough education, we also have education from infection control, they come up and do in-services, they also have it on the computer and also give handouts. (RN 1, Interview 3)"

### Skills

This domain describes the skills and competencies to perform HH and the procedural ability to use the EMS. Again, the data reveal a strong representation of this domain by nurse participants who stated that they have the skills required for performing HH and using the system.

"It’s not difficult at all. You have to use sanitizer or water and soap to wash your hands. No, not difficult at all, because we are always having in-services and computerized Q & A that we do from time to time and other sort of information around. (RN 2, Interview 4)"

Administrators however, perceived the skill level of the nurses as a potential limitation. The following quote demonstrates this discrepancy:

"Yes, I think they have some of those skills. It is just hard to focus on it. (ADM 1)"

### Social/professional role and identity

The third domain describes the nurses’ role and identity in regards to performing HH and the EMS system. Nurses and administrators clearly understood the purpose of the system and thought positively about the credibility of the EMS. Both groups describe the system as supporting nurses with their professional identity. The social and group norms are demonstrated by these quotes:

"This [the electronic monitoring system] is going to be very helpful, we have to focus it correctly and to the organization. (ADM 2)"

"I think we will be more aware and more mindful of stopping and taking the time to hand washing before we move on, and when we come in and out of the room. It is kind of drilled in our head now. (RN 3, Interview 5)"

### Beliefs about capabilities

This domain describes nurses’ perceived self-efficacy and control as potential barriers or facilitators to perform HH and use the EMS:

"I think it will increase [HH]…it will prompt us to perform HH more differently along with the education we get. I think it will lead us in the right direction. (RN 3, Interview 5)"

"Part of providing safe care, which is one of the standards, is to make sure that your hands are clean when you go to the patient. (RN 2, Interview 4)"

Administrators accordingly described the nurses’ beliefs about capabilities, yet added some difficulties within the healthcare environment that could potentially interfere with the use of the system. In other words, both groups perceived nurses to have behavioral control, yet administrators listed external difficulties and barriers that would limit nurses’ ability to perform proper HH:

"I am a great believer that staff can perform at a very high level but unfortunately, there are other things that come up in that particular moment that may take their mind away from doing what they know is best practice. (ADM 2)"

### Beliefs about consequences

The next domain describes nurses’ beliefs about the consequences of HH and using the EMS as potential barriers or facilitators. In the interviews, nurses focused consistently on the immediate outcome, that is, responding to the reminder signal to perform HH.

"I think [the EMS] will be very helpful and it will be a good reminder, make people aware of how often they wash their hands and the importance of washing their hands. (RN 1, Interview 3)"

"It will impact everyone and it’s going to be HH on a higher level. (RN 5, Interview 7)"

In contrast, administrators did not discuss the immediate consequences of the EMS but talked about the longitudinal aspect of the EMS data and the subsequent evaluative component for comparing nurses’ performance. Administrators also described the contingencies of the EMS, the benefits of using the system outweighing the costs, and the reinforcement and/or rewards when nurses did not perform as expected.

"I think with any system like this, at the beginning, it is going to be very confidential and perhaps down the line it may be reported to managers, and that is always a concern for the staff. (ADM 3)"

Only the administrators discussed the cost associated with increased HH compliance and the effect of the system that might lead to poor implementation of the EMS.

### Motivation and goals

This domain explores the barriers or facilitators to the motivations and goals to practice HH and use the EMS system. This domain refers to how high a priority the target behaviour is for the participant. Nurses concentrated foremost on their personal safety and their families’ safety as a source of motivation to perform HH:

"Since we had SARS, people are very, very conscious about what they can take home to their family, so they are very, very conscious. (RN 1, Interview 3)"

Interestingly, only one of the nurses mentioned patient safety when discussing the intention to perform HH or use the system initially. Administrators, on the other hand, did not discuss nurses’ personal safety as a motivator but believed that nurses would display some intrinsic and external motivators to perform HH, such as professional commitment, individual and unit incentives, and personalized goal setting:

"Incentive is good for a group who want to do well. There is no incentive for the others who draw back and do nothing, depends on the individual and the group. (ADM 3)"

"… even with results from infection control, it is always shared with staff and they are proud to hear what the results are and it’s just going to be ongoing with continuous feedback and enhance the hand hygiene. (ADM 2)"

The emphasis on a different characteristic within the domain of motivation and goals suggests that the implementation of the EMS will need to address these differing perspectives.

### Memory, attention, and decision processes

This domain explores memory, attention, and decision processes as barriers or facilitators in regards to influencing nurses’ decisions to wash hands and use the EMS system. As optimal HH is considered best practice in nursing practice, it would be assumed that nurses perceive HH as a routine procedure, a task they usually carry out before entering or after leaving a patient room. Nurses supported this assumption by clearly stating that they are constantly aware of the need to wash their hands, as demonstrated by these quotes:

"I am consciously thinking about [performing HH], especially when you are in a room with four people…we don’t want to go back and forth and you try to be more aware, or when you are coming out of the room, you try to make sure you wash before going in. (RN 3, Interview 5)"

"…I see the pump right there in the hall, so once I see it, I use it and go into the room and when I come out, I look for it and use it again …. (RN 3, Interview 5)"

Conversely, administrators stated that the staff do not remember to focus on performing HH and do not have the decision processes in place to judge whether HH is necessary or not.

"I think they don’t have time and I think they don't regard it seriously. (ADM 3)"

This interesting disparity calls for a need to collect data on the actual attention and decision processes of the nurses during the intervention implementation of the EMS.

### Environmental context and resources

This domain explores the availability and accessibility of resources to perform HH and use the EMS system as a facilitator to enhance HH and the EMS intervention. Both administrators and nursing staff felt there were adequate resources available, and accessibility and functionality of the resources were not deemed problematic.

"When I worked in long-term care there were no sinks in any of the rooms and now, you can’t go far out of a room without finding one. (ADM 3)"

"I think there are enough [resources] that are in place where we can easily access it. (RN 3, Interview 5)"

The above quotes indicate that this domain is relevant, yet not problematic, to the implementation of the EMS.

### Social influences

This next domain explores social influence as a potential barrier or facilitator in improving HH and using the EMS. Nurses and administrators had a very different perspective on the relevance of social influence. Administrators assumed that social support by peers, administrators, other professional groups, or patients would facilitate HH compliance. Furthermore, they anticipated that strong performers would act as role models and leaders to invite weaker performers to increase their HH performance. Teamwork and group cohesion will be a strong support to enhance the implementation of the EMS. Administrators also perceived that social support and teamwork would be utilized in a positive manner to help everyone achieve the goal of enhanced HH.

"I think that there are some staff that really get it and there could be a peer support system. (ADM 3)"

"When you have a role model that already thinks so positively of them [nurses], then they want to follow that type of role model. (ADM 2)"

Conversely, nurses described the social influences as a deterrent to implementing the system. Nurses were not interested in group conformity or social comparisons. Rather, they focused on their own behavior as negative social influence, potentially causing inter-group conflict. Nurses were focused on improving their individual performance and did not want to act as role models or leaders. They would rather leave poor performers to their own devices.

"…people do watch other people…. people would not like to talk or work with other people that did not wash their hands, so they are going to shun that person. (RN 1, Interview 3)"

"…once you say something you make them feel like you are watching them, looking for some mistakes, and of course, they’re going to retaliate. They’re going to do the same thing to you. And then it’s just going to escalate and then you’re going to have problems. It is good to tell them, I understand that, but there are consequences. (RN 6, Interview 9)"

### Emotion

The domain of emotion delves into any stress, fear, burnout, or emotional responses (positive or negative) that could be caused by performing HH and using the EMS acting as potential barriers or facilitators to enhancing HH. The authors note that this domain might appear to overlapping with the domain on the beliefs about consequences. To avoid any confusion, the authors strictly adhered to the theoretical domains discussed in the interviews and reported the results as such.

The EMS records or prompts nurses to comply with each opportunity for HH. Given a current HH compliance rate of below 40%, it is anticipated that nurses will have to double their HH activity, potentially causing a cognitive overload or even physical tiredness. The administrators seemed to be well aware of this risk, claiming that some of the HH performance measures cause frustration or a negative emotional response in staff:

"Guidelines could drive them crazy. (ADM 2)"

Nurses described negative emotions in relation to their own and families’ safety, although this was not directed towards the EMS or the HH practice.

"Fear and safety, you know because when we are here and we are more prone to anything that is here, then you don’t want to bring home flu to anyone else. (RN 3, Interview 5)"

### Behavioral regulation

This domain describes the behavioral regulation, that is, the preparatory steps needed to perform enhanced HH and use the EMS, as a barrier or facilitator. Within this domain, nurses described the importance of individual feedback and self-monitoring in order to increase their performance. Nurses reported being in favor of meeting with a mentor on a one-to-one basis to discuss goals and targets and to identify priorities and analyze performance data to support improvement:

"At the individual level, reminders. I think at the organizational level we are not doing too bad because we are doing a lot of teaching, so you know, it remains ongoing. We will get to a point where it will be at a higher level. (RN 3, Interview 5)"

Administrators however, believe that this behavioral regulation might be a barrier in the implementation of the EMS. As one of the administrators describes,

"I don’t think they [nurses] understand the importance. I think it still goes back to the education and we don’t have a lot of infections here so, why should I bother to get it? They also haven’t had patients ask them, ‘Have you washed your hands before you touch me?’ (ADM 1)"

### Nature of behaviours

This last domain depicts the nature of the proposed behaviour, that is, improved HH compliance, as a facilitator in regards to the current HH practice and the use of the EMS system. According to nurses, HH is currently being practiced as a routine, relying on habit. Yet, research also indicates that this “routine is followed in less than half of the situations where HH actions are required. The proposed HH behavior will not be any different than the current practice, however, the behavior will need to be performed more frequently. Nurses were aware that, as a group, the existing behavior needed to be practiced more often and that the EMS will prompt this new behavior:

"It maybe varies with other team members, yes, but with nursing, I think it is more on the table to keep your hands clean when going to another patient. Take your glove off and wash your hands. And I think it is because of all the education sessions, reminders like the [EMS]. (RN 1, Interview 3)"

Administrators supported this response as well but indicated that increasing the frequency of the proposed behavior might be more difficult than anticipated:

"…you have to build in sustainability and accountability, right…And the accountability can’t always lie with the manager. The staff has to be accountable, but how are we going to monitor it to make sure it is sustained? Because it falls off real quick. (ADM 3)"

## Discussion

This study used the TDI [26] to explore barriers and facilitators to current HH practices and the implementation of a new EMS to improve HH practice. Semistructured in-depth interviews were conducted that compared perceptions of nurses and administrators. In this study, facilitators and barriers to current HH practices and the implementation of the EMS intervention ranged from the individual level to organizational and social contexts. For the majority of the theoretical domains, differences in responses were noted between the administrators and the nurses. These differences provide insight into considerations for how the intervention should be implemented, as directed by the knowledge-to-action cycle [22].

Knowledge was perceived both as a barrier and facilitator by the participants. Nurses believed they already had sufficient knowledge about HH, whereas the administrators perceived that the nurses did not; therefore, the lack of knowledge acted as a barrier to current HH practices and the implementation of the EMS. This discrepancy will need to be addressed in the implementation plan for the EMS intervention. If nurses believe they have the knowledge and awareness as to how and when to perform HH, competency testing and educational sessions to update nurses’ knowledge and skills will not be viewed as useful. Rather, a focus on understanding why nurses decide to perform or skip HH actions may be of greater importance for the successful implementation of the EMS. For example, some of these reasons to perform or neglect HH could be explored during weekly and monthly educational sessions with the participants, rather than just focusing on a discussion of actual HH compliance rates [18].

One of the biggest discrepancies between the two groups was related to the beliefs about consequences domain. Administrators clearly indicated the potential consequences of using the EMS in the long term as a potential barrier, whereas nursing staff only perceived the immediate outcomes of the system. The lack of nurses’ focus on long-term outcomes might indicate that nurses do not realize the capabilities of the system, a problem easily corrected by providing additional information in the recruitment session or during the individualized educational sessions [18]; it might also point to a more serious issue based in a lack of ongoing commitment to improve practice. This phenomenon has been reported by other researchers analyzing quality improvements in nursing practice [32] and results from the current practice development movement focusing on immediate reportable outcomes and the lack of a systematic approach to the practice change. As a result, nurses act upon the immediate demand to change practice, yet they do not perceive the change in practice to be necessary nor valuable [32]. In order to create a sustainable HH practice change, it will be necessary to continuously explore and address individual nurses’ development and empowerment during the intervention phase.

Another major difference was in the social influences domain. Nurses did not describe social influences, such as the opinion or care practice of colleagues, as an enabler to implementation of the EMS. Nurses were focused on improving their individual performance and preferred to stay away from poor performers as opposed to creating a supportive network to enhance HH practice. The individuality of nursing performance has previously been described in the nursing literature [33]. Despite careful evidence on appropriate levels of HH and staff performance, [11,33] it appears that this knowledge was not translated to staff and administrators in this practice setting. Both administrators and nursing staff in this study and others did not know how often staff perform HH or the level of performance at the unit or facility level, nor do they know the acceptable HH compliance rates [34]. When developing the implementation plan, the integration of anonymous group data in the individual performance reports and the discussion of these data in the educational sessions with the individual participants might create a heightened awareness of one’s own performance compared to others [18,22,35]. The apparent differences between administrators and nurses in regards to several barriers and facilitators toward HH points to an intervention implementation strategy that will need to focus on the provision of support and mentoring for the nurses, rather than solely improving the frequency of HH [18,35].

Based on the data, the next step in the EMS intervention implementation needs to consist of identifying behavior-change techniques that target these specific determinants, such as self-monitoring of performance, graded goal setting and improving HH practice, and modeling and demonstration of behaviors by others [20-22,35]. Norms can be addressed by modeling/demonstration of behaviors by others; social processes of encouragement, pressure, and support; and prompts, triggers, cues [35,36]. Another behavior-change technique that will be considered during the intervention implementation is the use of the EMS data to create “social influences in order to enhance the uptake [36]. Weekly individual performance data reports will include a graph that indicates other participants’ overall compliance data to compare personal data against the levels in the group. During individual meetings with the participant, specific information on how to increase HH, various techniques for increasing HH (prompts, triggers, cues), and suggestions on how to change the behavior will be provided [18].

Limitations identified in this research include potential social desirability bias, the use of a simple coding scheme, and the small sample from one hospital unit. Further research is required to validate and refine this theoretical framework and the coding procedure [24]. Specific areas of interest for this research are related to identifying the most important determinants or behaviour-change domains, so as to guide the selection of which determinants to address and/or which of the techniques to adopt when implementing an intervention. It would also be beneficial to explore the relationship between the domains. If the awareness or skills are improved, would this change affect beliefs or capabilities? Lastly, it is important to note that despite the overall agreement within the nurses’ sample, the responses represent perceptions and not actual skills or knowledge. This was clearly demonstrated when all nurses claimed they had sufficient knowledge of HH guidelines, yet there was a clear gap in HH knowledge when asked specifically about HH skills. This suggests that relying on only perceptions is not sufficient and could negatively impact the implementation of the intervention.

### Significance

HAIs affect many patients and often result in suffering and deaths. Consistent HH can reduce these infection rates by up to 50%, yet healthcare staff often fail to perform HH. Several attempts have been made to improve compliance, without significant sustained increases. Novel approaches combined with a clear understanding of how to change nurses’ behaviour are required to improve HH compliance.

This work is an essential first step towards identifying factors affecting nurses’ behavior change associated with HH compliance. Findings of this study will guide the identification of optimal strategies to enhance adoption of “best HH practice by nurses working in different practice environments and will inform the subsequent intervention study [22]. The end goal of the intervention study is to enhance and sustain improved HH compliance and, ultimately, improve the safety and health of patients receiving care.

Findings of this study will lead to a better understanding of the effect of an intervention employing a monitoring system on HH compliance and will be instrumental in providing evidence to support intervention design and interpretation and decisions made by policy makers around HH practices. By enhancing and understanding factors underlying HH behaviour, this research will ultimately contribute to safer and better-quality care for patients. This study also contributes to the knowledge translation literature in that the knowledge generated from this work will provide a structured approach to developing an implementation plan with a better understanding of how to enhance healthcare professionals’ behaviours in complex interventions.

## Conclusion

This qualitative study demonstrated the value in using psychological theories commonly used in knowledge translation to explore barriers and facilitators to current HH practices and when implementing an intervention to improve HH practice. Perspectives of administrators and nurses were compared for their relevance to HH and the implementation of an EMS to enhance HH. The results provide a better understanding of nurses’ behaviour-change processes of the EMS intervention and will be integrated to refine the implementation plan.

Rigorous exploration of theoretical domains of behavior change acting as potential barriers and facilitators will not only lead to appropriate selection of the intervention components but will also promote the adaptation of the knowledge to the local context. In respecting these simple, yet essential, steps in the process of integrating knowledge into practice before actual intervention design and implementation, it is hypothesized that interventions will be more successful and acquired knowledge more sustainable.

## Competing interests

VMB received funding from Toronto Rehabilitation Institute for the development and testing of the EMS. GRF is Vice President of Research at Toronto Rehabilitation Institute and is the principal investigator on the EMS project. The other authors declare they have no competing interests.

## Authors' contributions

VMB was responsible for the research idea and project management and led the interview design and data analysis. She wrote the first draft of the paper and subsequent redrafts. All authors contributed to the development of the research objectives and methods and to the writing of the paper. JHL supported the data collection and helped analyze data. All authors read and approved the final research protocol and manuscript. All authors contributed equally to this study.

## Authors' information

VMB is currently the CIHR Schlegel Chair for Enhanced Seniors' Care, Schlegel-University of Waterloo Research Institute for Aging (RIA) & School of Health & Life Sciences and Community Services, Conestoga College Institute of Technology and Advanced Learning. At the time of the study, VMB was a postdoctoral fellow at Toronto Rehabilitation Institute. SBJ is the Toronto Rehabilitation Institute Chair at the University of Toronto.
